# Supplementary material for: Divergent roles of DNA methylation, TRIM28, and p53 surveillance in human embryonic and trophoblast stem cells
Source: bioRxiv. 2025 Oct 20:2025.10.19.683051. Preprint. [Version 2] doi: 10.1101/2025.10.19.683051 (PMC12633553; doi:10.1101/2025.10.19.683051)
Supplement: Supplement 7 [file media-7.pdf]

Supplementary Table captions

Table S1. Replicate information and mapping statistics for all next generation sequencing libraries generated in this manuscript.

Table S2. All datasets mined in this manuscript

Table S3. For each LTR class, the number of TRIM28 ChIP-seq peaks, the percent methylation, the percent methylation relative to surrounding sequence, and the ATAC-seq enrichment in the cell types indicated. Also shown is RAD analysis showing ratio of (upregulated in hTSC/upregulated in hESC) for genes within 50kb of an element of this class.

Table S4 All differentially expressed gene calling and RPKM values for RNA-seq conducted in the course of this manuscript.

Table S5 List of all TRIM28-bound LTR transposon within 50kb of a gene upregulated upon TRIM28 knockout in hTSCs.

Table S6 Analysis to identify methylation-regulated genes, showing gene sets identified in Figure S4A.
