# Supplementary material for: Divergent roles of DNA methylation, TRIM28, and p53 surveillance in human embryonic and trophoblast stem cells
Source: bioRxiv. 2025 Oct 20:2025.10.19.683051. Preprint. [Version 2] doi: 10.1101/2025.10.19.683051 (PMC12633553; doi:10.1101/2025.10.19.683051)
Supplement: 1 [file NIHPP2025.10.19.683051V2-supplement-1.pdf]

bioRxiv preprint doi: https://doi.org/10.1101/2025.10.19.683051; this version posted October 20, 2025. The copyright holder for this preprint (which was not certified by peer review) is the author/funder, who has granted bioRxiv a license to display the preprint in perpetuity. It is made available under aCC-BY 4.0 International license.

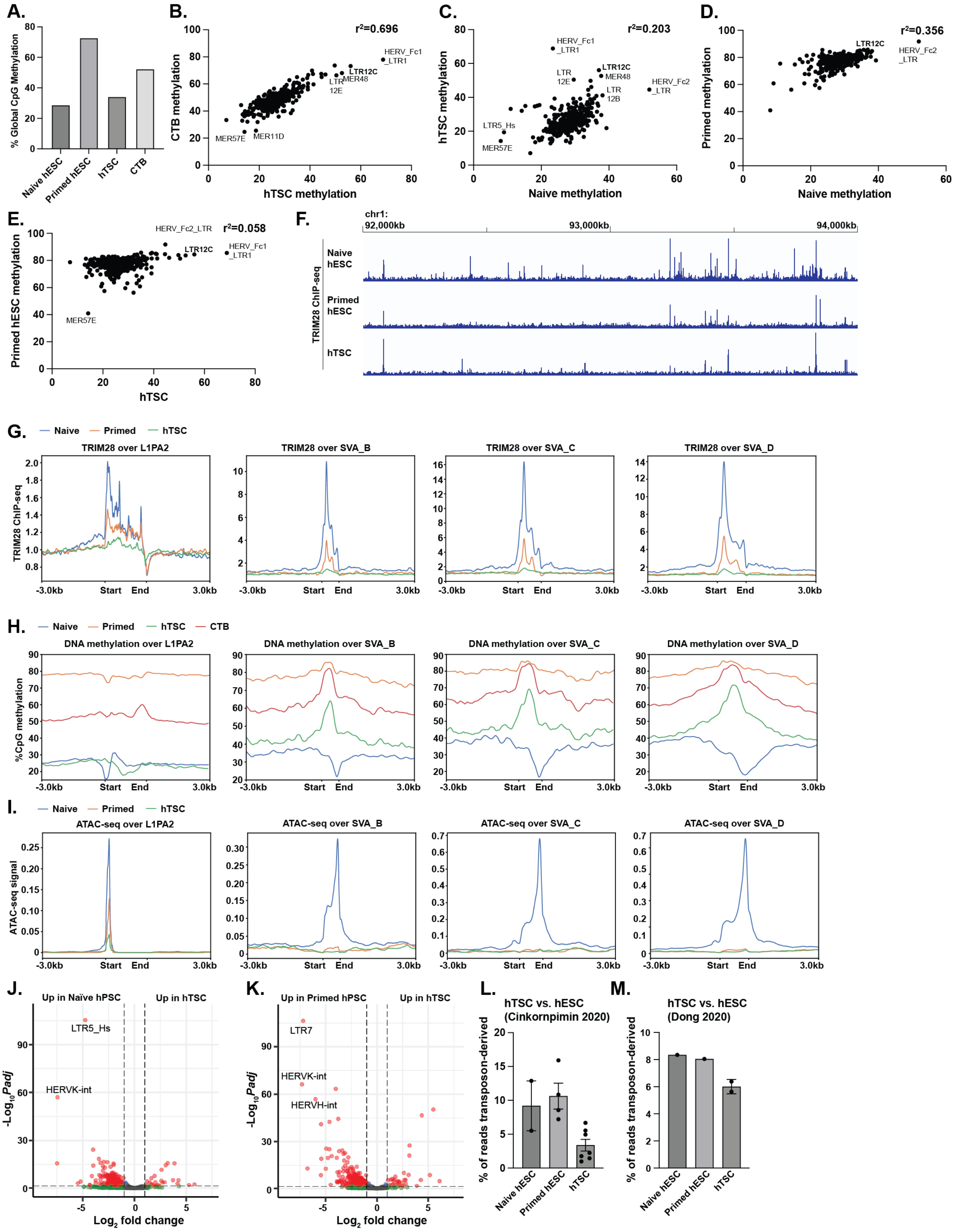

bioRxiv preprint doi: https://doi.org/10.1101/2025.10.19.683051; this version posted October 20, 2025. The copyright holder for this preprint (which was not certified by peer review) is the author/funder, who has granted bioRxiv a license to display the preprint in perpetuity. It is made available under aCC-BY 4.0 International license.

Figure S1. Distribution of TRIM28 and 5mC over transposons and transposon expression in in embryonic and trophoblast stem cells, related to Figure 1.
(A) Global %CpG methylation of cell types indicated.
(B - E) Average CpG methylation level of each LTR class in cell types indicated, with correlation coefficient calculated. Note high correlation of LTR methylation between CTB and hTSC.
(F) TRIM28 ChIP-seq signal over a 2MB region on chromosome 1. Note the presence of far more TRIM28 sites in Naïve hESC relative to Primed or hTSC.
(G) Metaplot of TRIM28 ChIP-seq signal over transposon classes indicated in naïve hESC, primed hESCs, and hTSCs.
(H) Metaplot of %CpG methylation over transposon classes indicated in naïve hESC, primed hESCs, and hTSCs.
(I) Metaplot of ATAC-seq signal over transposon classes indicated in naïve hESC, primed hESCs, and hTSCs.
(J) Volcano plot of expression of transposon classes in hTSC vs. naïve hESC.
(K) Volcano plot of expression of transposon classes in hTSC vs. primed hESC.
(L, M) Percentage of transposon-derived reads in cell type indicated for data source indicated.

bioRxiv preprint doi: https://doi.org/10.1101/2025.10.19.683051; this version posted October 20, 2025. The copyright holder for this preprint (which was not certified by peer review) is the author/funder, who has granted bioRxiv a license to display the preprint in perpetuity. It is made available under aCC-BY 4.0 International license.

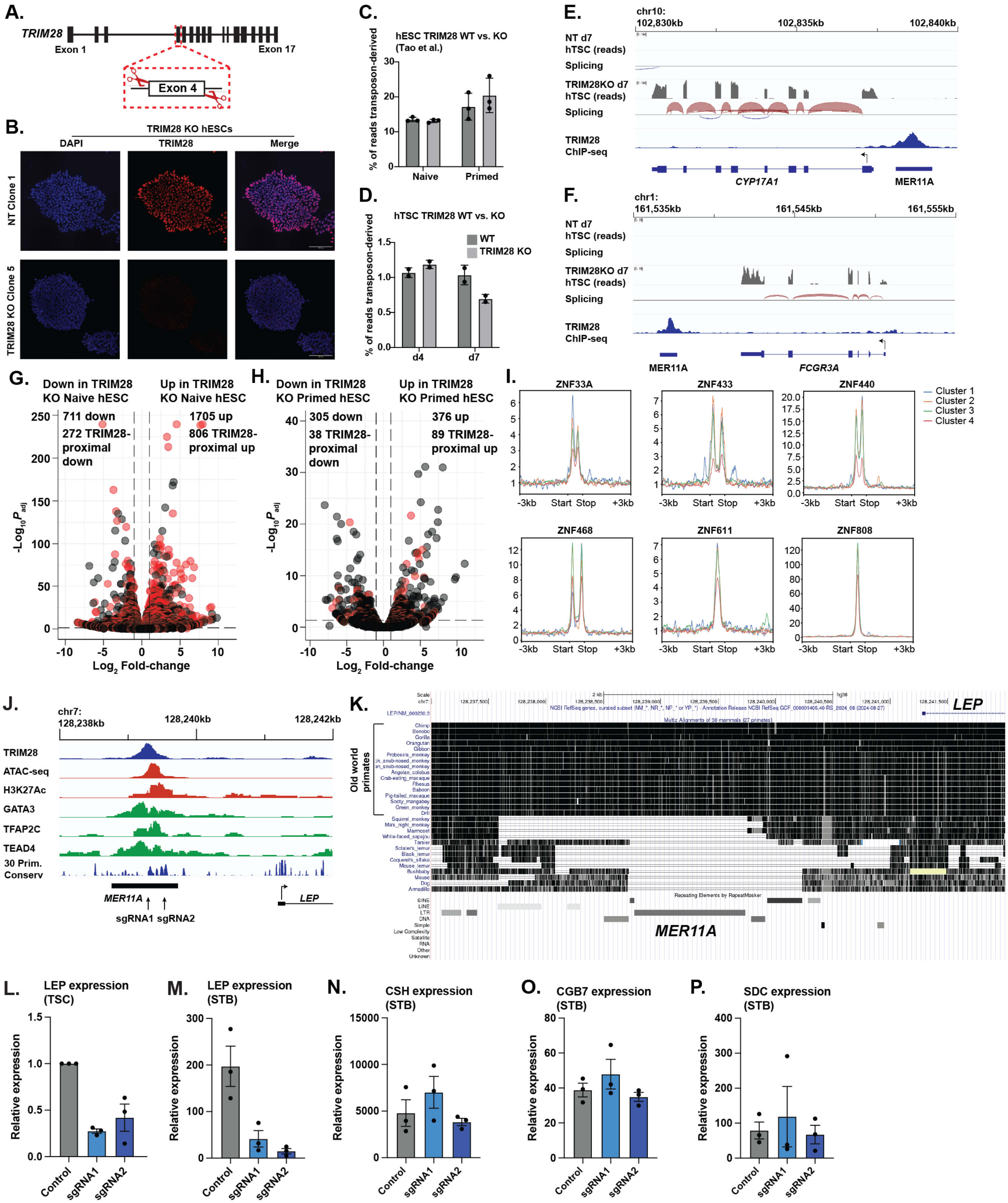

Figure S2. TRIM28 regulates adjacent genes by suppression of LTR transposons, related to Figure 2.

(A) Schematic of targeting of *TRIM28* locus.

bioRxiv preprint doi: https://doi.org/10.1101/2025.10.19.683051; this version posted October 20, 2025. The copyright holder for this preprint (which was not certified by peer review) is the author/funder, who has granted bioRxiv a license to display the preprint in perpetuity. It is made available under aCC-BY 4.0 International license.

(B) Immunofluorescent staining for TRIM28 in a TRIM28 KO hESC clonal line and a control clonal line nucleofected with TRIM28-targeting sgRNA or non-targeting sgRNA. hESC were stained with TRIM28 (red) and DAPI (blue). Pictures were taken at 20x and the scale bar measures 100 µm.
(C) Percentage of transposon-derived reads from control and TRIM28$^{-/-}$ naïve and primed hESCs. Data is derived from Tao et al. 2018.
(D) Percentage of transposon-derived reads from control and bulk TRIM28 d4 and d7 KO hTSC.
(E, F) RNA-seq and ChIP-seq plotted over the *CYP17A1* (E) and *FCGR3A* (F) loci in control and d7 *TRIM28* KO hTSCs. Note that while both genes are highly upregulated in the TRIM28 KO, there is no splicing of transcripts from the MER11A element to the genes.
(G, H) Volcano plot of TRIM28 KO vs. control naïve (G) and primed (H) hESCs. TRIM28-proximal (<50kb distance) genes are indicated in red.
(I) Metaplots of six KRAB-ZFN proteins over the four MER11A clusters identified in Figure 3F. Most show uniform enrichment over Clusters 1 - 3, but ZNF468 shows lower enrichment over the less heterochromatinized Cluster 1. Note that this ChIP-seq data is derived from ectopic expression of KRAB-ZFNs in 293T cells.
(J) ATAC-seq and ChIP-seq enrichment plotted over a 4kb region upstream of and including the *LEP* promoter. Sites of two sgRNA used for CRISPRi targeting of the locus are indicated.
(K) Conservation of region upstream of *LEP* in twenty-six primate and three non-primate mammals. Note that the MER11A element is only present in old world primates.
(L, M) Expression of *LEP* in hTSCs and STBs expressing sgRNA targeting sites indicated in (J) or a non-targeting control sgRNA. Expression is normalized to level in control hTSC.
(N – P) qPCR data showing expression of the STB markers *CSH* (N), *CGB7* (O) and *SDC* (P) in STBs expressing a CRISPRi system and the sgRNA indicated in (J). Note normal induction of these STB genes in all three samples, indicating the sgRNA had no general effect on STB differentiation.

bioRxiv preprint doi: https://doi.org/10.1101/2025.10.19.683051; this version posted October 20, 2025. The copyright holder for this preprint (which was not certified by peer review) is the author/funder, who has granted bioRxiv a license to display the preprint in perpetuity. It is made available under aCC-BY 4.0 International license.

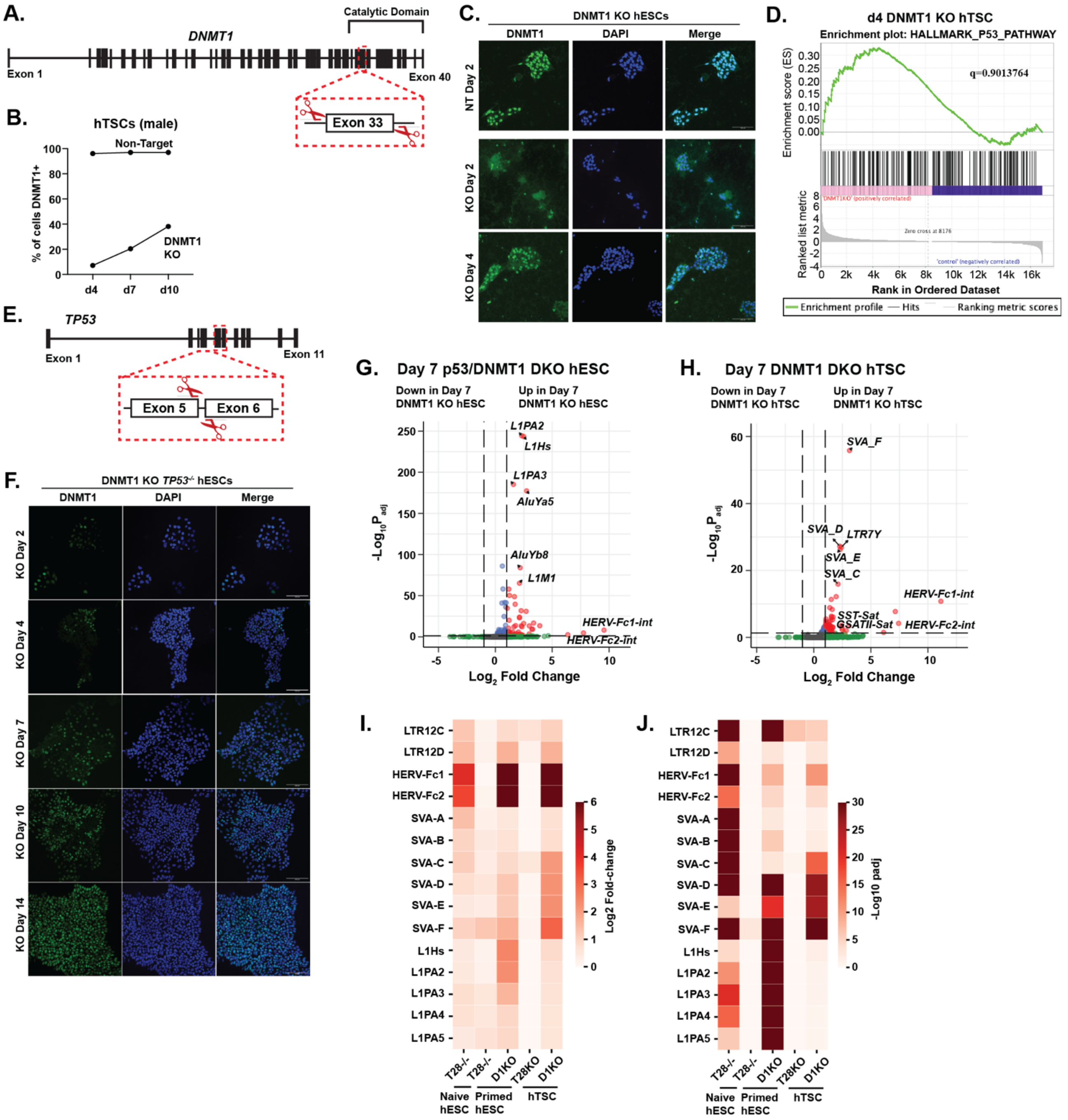

Figure S3. DNA methylation is essential for survival and transposon repression of hTSCs and hESCs, related to Figure 3

(A) Schematic of targeting of *DNMT1* locus.

(B) Percentage of CT29 hTSC positive for DNMT1 after nucleofection with CRISPR/Cas9 and sgRNA targeting DNMT1 (DNMT1 KO) or non-targeting sgRNA (Non-target). Rate of loss for the male CT29 line is similar to the rate of loss for the female CT30 line as shown in Figure 3A.

(C) Immunofluorescent staining for DNMT1 in a bulk population of hESCs 2 or 4 days after nucleofection with CRISPR/Cas9 and sgRNA targeting DNMT1. hESCs that underwent nucleofection with a non-targeting sgRNA are shown as a control. Note rapid loss of *DNMT1* deficient hESCs. hESCs were stained for DNMT1 (green) and DAPI at a magnification of 20x and the scale bar measures 100 µm.

bioRxiv preprint doi: https://doi.org/10.1101/2025.10.19.683051; this version posted October 20, 2025. The copyright holder for this preprint (which was not certified by peer review) is the author/funder, who has granted bioRxiv a license to display the preprint in perpetuity. It is made available under aCC-BY 4.0 International license.

(D) GSEA analysis comparing expression of P53 pathway genes in day 4 DNMT1 KO hTSCs and controls. No statistically significant difference increase is observed in the DNMT1 KO cells.
(E) Schematic of targeting of *TP53* locus.
(F) Immunofluorescent staining for DNMT1 in a bulk population of *TP53*$^{-/-}$ hESCs after nucleofection with CRISPR/Cas9 and sgRNA targeting DNMT1. hESC's that underwent nucleofection with a non-targeting sgRNA are shown as a control. Note slower loss of *TP53*$^{-/-}$ DNMT1 KO hESCs as compared with S2C. hESCs were stained for DNMT1 (green) and DAPI at a magnification of 20x and the scale bar measures 100 µm.
(G, H) Volcano plot of transposon classes upregulated in DNMT1 KO hESC (G) and hTSC (H).
(I, J) Fold change (I) and statistical significance of upregulation (J) for transposon class and cell type indicated. Note that elements such as SVAs and LINEs switch from negative regulation by TRIM28 in naïve hESCs to negative regulation by DNA methylation in primed hESCs and hTSC, in concert with the DNA methylation gain observed in Figure 1F-H and S1G-1.

bioRxiv preprint doi: https://doi.org/10.1101/2025.10.19.683051; this version posted October 20, 2025. The copyright holder for this preprint (which was not certified by peer review) is the author/funder, who has granted bioRxiv a license to display the preprint in perpetuity. It is made available under aCC-BY 4.0 International license.

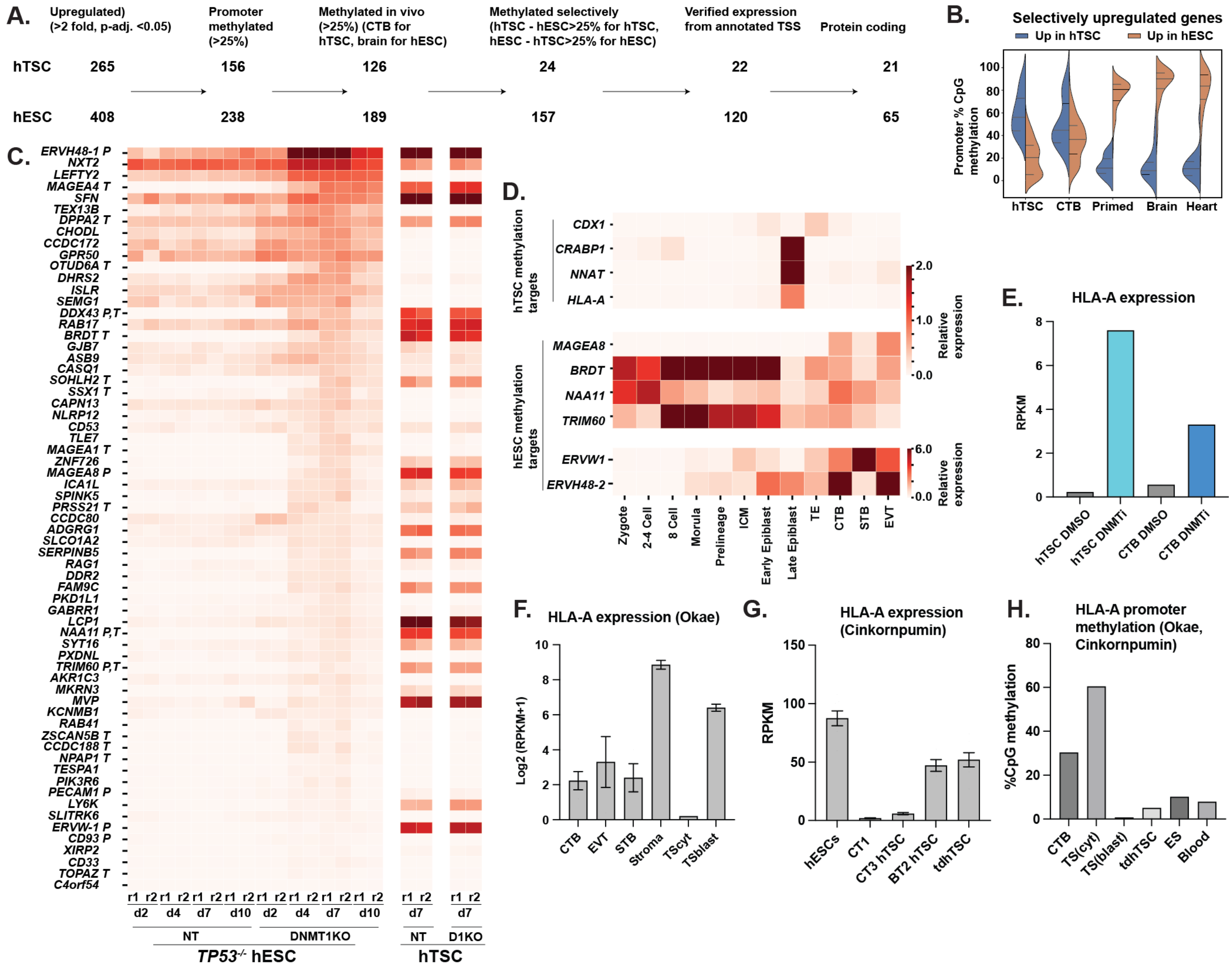

Figure S4. Regulation of developmentally important genes by DNA methylation in trophoblast and epiblast lineage, related to Figure 4.

(A) Schematic for identification of genes regulated by 5mC selectively in one lineage. Starting from the genes only upregulated in one set in (A), required >25% promoter methylation (promoter: -500bp to 300bp), >25% methylation in placental or somatic tissue, methylated selectively in one tissue but not both. We next confirmed expression from the indicated promoter on Integrative Genome Viewer (IGV) to confirm a set of 5mC-regulated transcripts. The subset that are protein coding is finally indicated.

(B) Split violin plot indicating promoter DNA methylation of the 22 hTSC-specific and 120 hESC-specific transcripts.

(C) Heatmap showing expression of protein coding genes repressed by 5mC in hESCs in cell types indicated. Genes specifically expressed in placental (P) and/or testis (T) as determined by Human Protein Atlas are specifically labelled.

(D) Single cell analysis of expression levels of representative hTSC and hESC methylation target genes in human embryos from zygote to epiblast and placental cells.

(E) Expression of HLA-A in control and DNMT1i-treated hTSC and CTB.

(F) HLA-A expression in tissues indicated, as well as placenta-derived (TScyt) and blastocyst-derived (TSblast) hTSC.

(G) HLA-A expression in cell type indicated. tdhTSC refers refers to hTSC generated by transdifferentiation from hESC.

bioRxiv preprint doi: https://doi.org/10.1101/2025.10.19.683051; this version posted October 20, 2025. The copyright holder for this preprint (which was not certified by peer review) is the author/funder, who has granted bioRxiv a license to display the preprint in perpetuity. It is made available under aCC-BY 4.0 International license.

(H) HLA-A promoter methylation in cell type indicated. All data are from Okae 2018, except for TS(transdiff) which is from Cinkornpumin 2020. Note concordance of hypomethylation and leaky expression of HLA-A in blastocyst-derived or transdifferentiated hTSC.

bioRxiv preprint doi: https://doi.org/10.1101/2025.10.19.683051; this version posted October 20, 2025. The copyright holder for this preprint (which was not certified by peer review) is the author/funder, who has granted bioRxiv a license to display the preprint in perpetuity. It is made available under aCC-BY 4.0 International license.

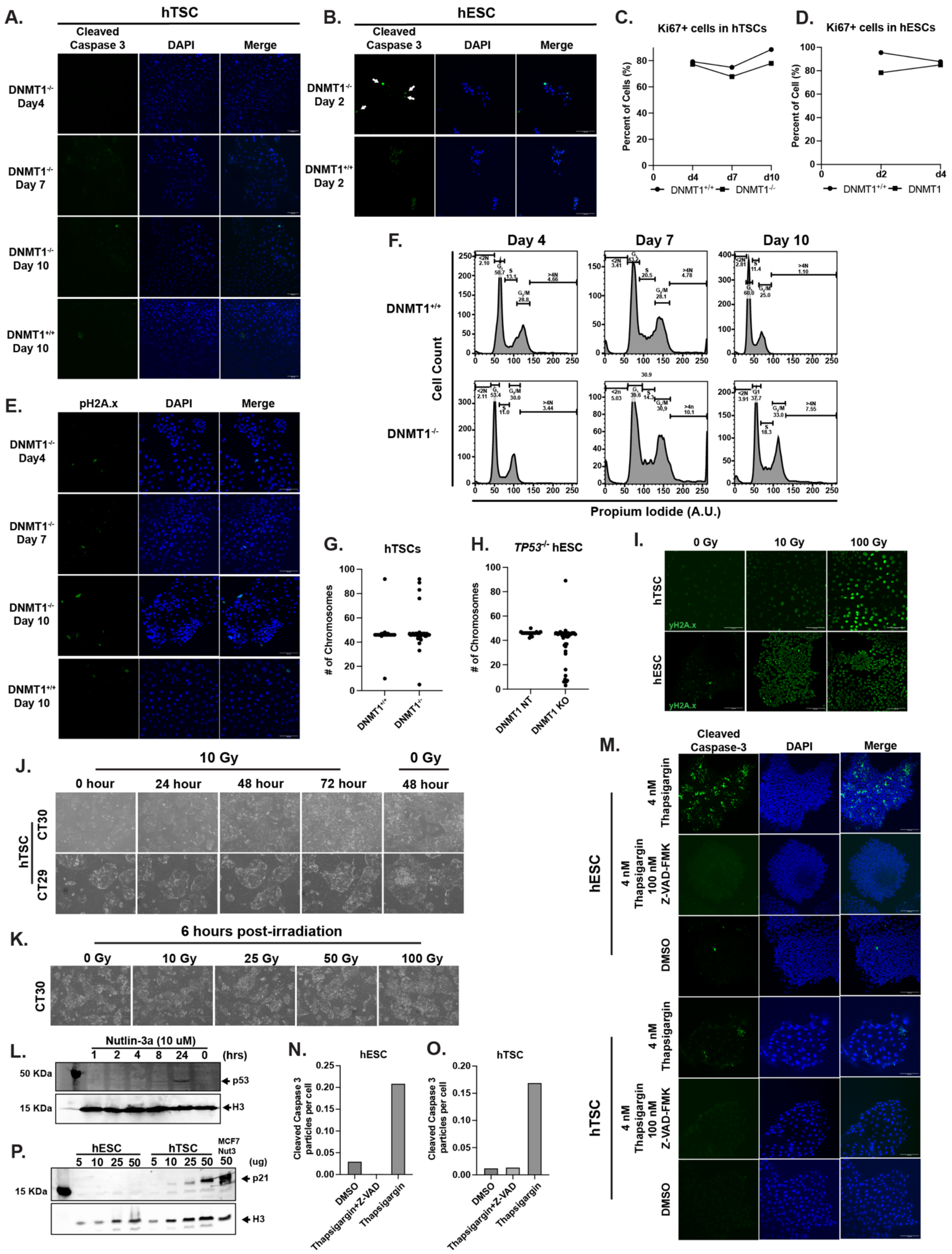

bioRxiv preprint doi: https://doi.org/10.1101/2025.10.19.683051; this version posted October 20, 2025. The copyright holder for this preprint (which was not certified by peer review) is the author/funder, who has granted bioRxiv a license to display the preprint in perpetuity. It is made available under aCC-BY 4.0 International license.

Figure S5. hTSCs have a diminished response to DNA damage despite active p53, related to Figure 5.
(A,B) Immunofluorescent staining for active apoptosis in (A) hTSCs and (B) hESCs after DNMT1 ablation. *DNMT1*$^{+/+}$ and *DNMT1*$^{-/-}$ hTSCs were stained for cleaved caspase 3 (green) and DAPI (blue). Note the lack of active cleaved caspase 3 signal in *DNMT1*$^{-/-}$ hTSCs and arrows showing enrichment of positive cleaved caspase 3 in *DNMT1*$^{-/-}$ hESCs. Pictures were taken at 20x magnification and the scale bar measures 100 µm.
(C,D) Quantification of Ki67 positive cells in *DNMT1*$^{+/+}$ and *DNMT1*$^{-/-}$ (C) hTSCs and (D) hESCs from immunofluorescent pictures.
(E) Immunofluorescent staining of *DNMT1*$^{+/+}$ and *DNMT1*$^{-/-}$ hTSCs stained for yH2A.x (green) and DAPI (blue). Pictures were taken at 20x magnification and the scale bar measures 100 µm.
(F) Flow cytometry of *DNMT1*$^{+/+}$ and *DNMT1*$^{-/-}$ hTSCs stained with propidium iodide to measure DNA content. Note the increase in polyploid cells in *DNMT1*$^{-/-}$ hTSCs in Day 7 and Day 10 samples.
(G-H) Swarm plots quantification of chromosomal counts in Figure 5C.
(I) Immunofluorescent staining of wild-type hTSCs and hESCs exposed to 0, 10 or 100 Grays of ionizing radiation. Cells were stained for yH2A.x (green). Note the increasing intensity of yH2A.x signal with increased exposure to ionizing radiation. Pictures were taken at 20x magnification and the scale bar measures 100 µm.
(J) Timelapse brightfield picture of hTSCs, CT30 (female) and CT29 (male), exposed to 10 Grays of ionizing radiation. Pictures were taken every 24 hours to capture growth of cells after radiation exposure. Control hTSCs were not exposed to ionizing radiation (0 Grays) and seeded at the same density as radiation-treated cells. Pictures were taken at 4x magnification.
(K) Brightfield pictures of hTSCs exposed to 0-100 Grays of ionizing radiation. Pictures were taken 6 hours post-radiation exposure. Note the tolerance of hTSCs to 100 Grays of ionizing radiation. Pictures were taken at 4x magnification and the scale bar measures 750 µm.
(L) Western blot images of p53 enrichment in wild-type hTSC treated with 10 uM Nutlin-3a for 0 to 24 hours. 24 hours of Nutlin-3a treatment was required for positive p53 signal.
(M) Immunofluorescent staining of hESCs and hTSCs exposed to either Thapsigargin, Thapsigargin and Z-VAD-FMK or DMSO. Cells were stained with cleaved caspase 3 (green) and DAPI (blue) to measure active apoptosis. Note enrichment of cleaved caspase 3 signal in Thapsigargin treated cells and the lack of signal when apoptosis is blocked with Z-VAD-FMK. Pictures were taken at 20x magnification and the scale bar measures 100 µm.
(N, O) Quantification of images in M, showing cleaved-caspase3 particles per cell in (N) hESCs and (O) hTSCs.
(P) Western blot analysis hESCs and hTSCs expression of p21 protein levels. Protein lysates were stained for both p21 and H3. Positive signal included MCF cells exposed to UV radiation. Increasing protein lysates from 5 to 50 ug were added per samples.

bioRxiv preprint doi: https://doi.org/10.1101/2025.10.19.683051; this version posted October 20, 2025. The copyright holder for this preprint (which was not certified by peer review) is the author/funder, who has granted bioRxiv a license to display the preprint in perpetuity. It is made available under aCC-BY 4.0 International license.

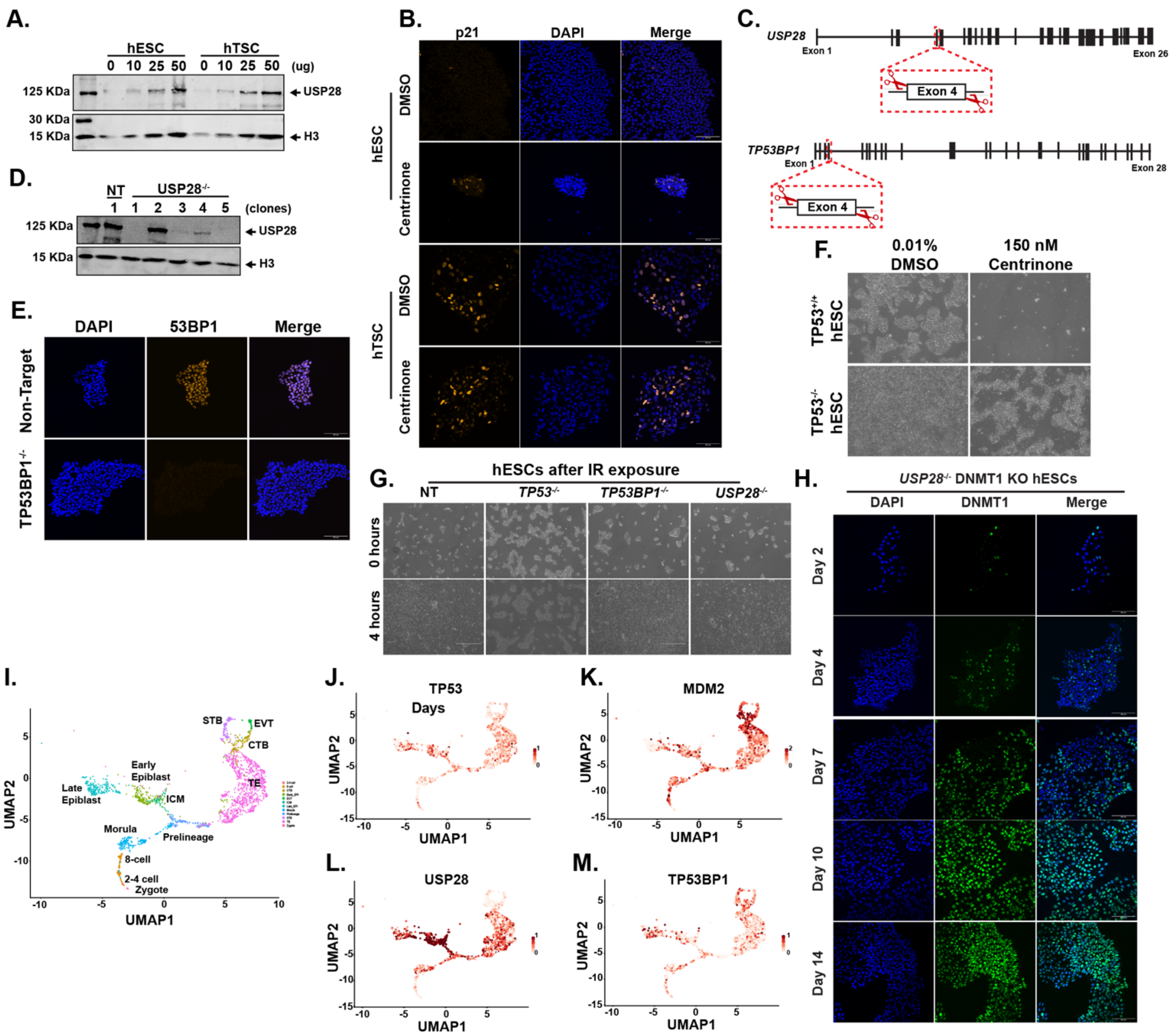

Supplemental Figure 6. The mitotic surveillance pathway is activated in DNMT1 knockout cells, related to Figure 6

(A) Western blot analysis to USP28 protein level in hESCs and hTSCs. Protein lysates input was added in increased concentration from 5 ug to 50 ug. Lysates were stained for both USP28 and H3.

(B) Immunofluorescent staining of mitotic surveillance activation after centrinone treatment in hESCs and hTSCs. Cells were treated with 150 nM centrinone or DMSO for 48 hours and stained for p21 (orange) and DAPI (blue). Pictures were taken at 20x magnification and the scale bar measures 100 µm. Note activation of p21 in hESCs exposed to centrinone and constant expression of p21 in hTSC.

(C) Schematic of CRISPR-Cas9 strategy to target USP28 and TP53BP1 in hESCs. sgRNA guides (red scissors) were designed to flank Exon 4 to generate frameshift mutation.

(D) Western blot analysis of hESC clones after USP28 ablation. Clones 1 and 5 were chosen for subsequent experiments.

(E) Immunofluorescent staining of hESCs clones after 53BP1 ablation. hESCs were stained for 53BP1 (orange) and DAPI. Note lack of 53BP1 expression in the *TP53BP1*$^{-/-}$ hESCs. Pictures were taken at 20x magnification and the scale bar measures 100 µm.

bioRxiv preprint doi: https://doi.org/10.1101/2025.10.19.683051; this version posted October 20, 2025. The copyright holder for this preprint (which was not certified by peer review) is the author/funder, who has granted bioRxiv a license to display the preprint in perpetuity. It is made available under aCC-BY 4.0 International license.

(F) Brightfield pictures of *TP53*$^{+/+}$ and *TP53*$^{-/-}$ hESCs after exposure to 150 nM centrinone. Cells were seeded at 100K in a 6-well plate and followed over the course of 4 days. Note tolerance of *TP53*$^{-/-}$ hESCs to centrinone treatment. Pictures were taken at 4x magnification.
(G) Brightfield pictures of control, *TP53*$^{-/-}$, *USP28*$^{-/-}$, and *TP53BP1*$^{-/-}$ hESCs upon treatment with 10 Grays of ionizing radiation. Pictures were taken at 4x magnification and the scale bar measures 750 μm.
(H) Immunofluorescent staining of *USP28*$^{-/-}$ hESCs after DNMT1 ablation. Cells were stained for DNMT1 (green) and DAPI (blue). Pictures were taken at 20x magnification and the scale bar measures 100 μm.
(I) Re-analysis of single cell RNAseq from human embryos Seurat objects datasets from Zygote to Epiblast/CTB.
(J-M) Expression heatmap profiles of *TP53, MDM2, USP28* and *TP53BP1* in during human embryo development.
